# Comparison of robotic-assisted versus conventional laparoscopic surgery in colorectal cancer resection: a systemic review and meta-analysis of randomized controlled trials

**DOI:** 10.3389/fonc.2023.1273378

**Published:** 2023-10-26

**Authors:** Zhilong Huang, Shibo Huang, Yanping Huang, Raoshan Luo, Weiming Liang

**Affiliations:** The First Affiliated Hospital of Guangxi University of Science and Technology, Guangxi University of Science and Technology, Liuzhou, Guangxi, China

**Keywords:** robot-assisted colorectal surgery, laparoscopic-assisted colorectal surgery, colorectal cancer, randomized controlled trial, complication

## Abstract

**Introduction:**

There is still controversy on whether or not robot-assisted colorectal surgery (RACS) have advantages over laparoscopic-assisted colorectal surgery(LACS).

**Materials and methods:**

The four databases (PubMed, Embase, Web of Science and Cochrane Library)were comprehensively searched for randomized controlled trials (RCTs) comparing the outcomes of RACS and LACS in the treatment of colorectal cancer from inception to 22 July 2023.

**Results:**

Eleven RCTs were considered eligible for the meta-analysis. Compared with LACS,RACS has significantly longer operation time(MD=5.19,95%CI: 18.00,39.82, P<0.00001), but shorter hospital stay(MD=2.97,95%CI:−1.60,−0.33,P = 0.003),lower conversion rate(RR=3.62,95%CI:0.40,0.76,P = 0.0003), lower complication rate(RR=3.31,95%CI:0.64,0.89,P=0.0009),fewer blood loss(MD=2.71,95%CI:−33.24,−5.35,P = 0.007),lower reoperation rate(RR=2.12, 95%CI:0.33,0.96,P=0.03)and longer distal resection margin(MD=2.16, 95%CI:0.04,0.94, P = 0.03). There was no significantly difference in harvested lymph nodes, the time of first flatus, the time of first defecation,the time of first resume diet, proximal resection margin, readmission rates, mortalities and CRM+ rates between two group.

**Conclusions:**

Our study indicated that RACS is a feasible and safe technique that can achieve better surgical efficacy compared with LACS in terms of short-term outcomes.

**Systematic review registration:**

https://www.crd.york.ac.uk/prospero/, identifier CRD42023447088.

## 1 Introduction

Colorectal surgery is widely used worldwide for benign and malignant lesions, including colorectal cancer(CRC). Colorectal cancer is the third most common cancer worldwide with an estimated annual incidence of 10,000 worldwide and the second leading cause of cancer deaths (1). At present, epidemiological studies have shown that the incidence of colorectal cancer is also gradually increasing in young people (2). The management of CRC is multidisciplinary; Surgery remains the most effective treatment, however, it is only available for patients with early stage cancer, while chemotherapy, targeted therapy, immunotherapy, surgery, and radiation are commonly used for advanced CRC (3–6).

At present, colorectal resection is still the main treatment strategy for colorectal cancer. Decades of development have proved that laparoscopic surgery is feasible and effective in the treatment of CRC, which greatly improves patient outcomes and does not have negative effects in terms of oncology and safety, and is considered as the gold standard treatment for colorectal cancer (7–11).

Robotics has flourished in recent years and the development of robotic surgery is considered as a major innovation in modern medicine since it offers an alternative to surgical methods in different situations (12). Robot-assisted technology is also widely used in colorectal cancer surgery, where robots offer many advantages over laparoscopic-assisted colorectal surgery(LACS), such as three-dimensional vision, 7° wrist-like motion, tremor filtration, motion scaling, better ergonomics, and less fatigue. These technical advantages can help overcome the drawbacks of LACS, such as two-dimensional vision, limited flexibility, and tremor (13). But in terms of clinical efficacy, conclusions of previous studies were conflicting on whether or not robot-assisted colorectal surgery (RACS) have advantages over LACS. Some studies declared that laparoscopic surgery was more advantageous, providing a high quality of colorectal resection, minimizing the damage to the tissue and organs of the surrounding tissue (14–17). However, other studies reported that clinical outcomes of RACS were better than those of traditional laparoscopic surgery (18–22).

Systemic reviews and meta-analysis had been performed to compare RACS and LACS.A meta-analysis showed that the two methods had similar clinical outcomes (23), but other meta-analysis declared that RACS had advantages regarding surgical efficacy and morbidity compared to LACS (24, 25). However, due to the shortage of strict inclusion criteria, a large amount of low evidence level RACS studies such as retrospective studies was included in above studies, which might resulted in probably unreliable conclusions.

Therefore, we conducted a systemic review and meta-analysis inclusion of only randomized controlled trials with high level of evidence. Our study aimed to compare the efficacy and safety of RACS and LACS in the treatment of colorectal cancer. These results may help provide high level evidence to support patients and physicians in their choice of CRC surgery.

## 2 Materials and methods

### 2.1 Search strategy

This meta-analysis was reported in accordance with the Preferred Reporting Project for Systematic Review and Meta-Analysis (PRISMA) 2020 guidelines (26, 27). This study was registered at PROSPERO under registration number CRD42023447088.The databases of PubMed, Embase, Web of science, and the Cochrane Library were systematically searched for papers published up to July 21, 2023. The MeSH terms “colorectal tumor”,”rectal tumor”,”colon tumor”,”laparoscopic”,”robot” as well as “randomized controlled trial” the free word “robot” and other relevant keywords were used in the search. The details of the searching record in four databases were shown in **Supplement Tables 1**–**4**.

### 2.2 Inclusion and exclusion criteria

Search strategies are developed in accordance with the PICOS principles (28) and then screened according to inclusion and exclusion criteria. Inclusion criteria were as follows (1): a randomized controlled trial comparing RACS with LACS for the treatment of patients with colorectal cancer (2); full-text articles reporting at least one of the following outcomes: operative time, hospital stays, blood loss, number of harvested lymph nodes, time of first flatus, time of first autonomous urination, time of first defecation, time of first resume diet,proximal resection margin, distal resection margin, rates of conversion to other surgery, complication rates, reoperation rates and mortality. Exclusion criteria were (1): other types of articles, such as conference abstracts yearbook, case reports, publications, letters, meta-analyses, reviews, retrospective studies, pharmacological intervention, animal studies and protocols (2); The full text cannot be obtained (3); Data duplication (4); Data could not be extracted for meta-analysis.

### 2.3 Data extraction

The study was divided into two phases with two independent investigators (L.H. and S.H.) reading the title and abstract, and then reading the full text. Differences were resolved by inviting a third investigator (Y.H.). Data retrieved included first author’s name, year, country, sample size, intervention, control, male ratio, age, treatment, body mass index, outcome, operative time, hospital stays, blood loss, number of harvested lymph nodes, time of first flatus, time of first autonomous urination, time of first defecation, time of first resume diet, proximal resection margin, distal resection margin, rates of conversion to other surgery, complication rates, reoperation rates and mortality.

### 2.4 Risk of bias assessment

The risk of bias was assessed using the Cochrane Risk of Bias tool (29) by two independent reviewers(L.H. and S.H.),according to the following domains: random sequence generation, allocation concealment, blinding of participants and personnel, blinding of outcome assessment, incomplete outcome data, selective reporting and others bias. The controversial results were resolved by group discussion if there were discrepancies.

### 2.5 Statistical analysis

The selection duplicate removal of studies included was conducted using EndNote (Version 20; Clarivate Analytics). All analyses were performed using Review manager 5.3(Cochrane Collaboration, Oxford, UK). Continuous variables were compared using weighted mean difference (WMD) with a 95% confidence interval (CI). Relative ratio (RR) with 95% CI were used to compare binary variables. The medians and interquartile ranges of continuous data were converted to the mean and standard deviation. Statistical heterogeneity between included studies was calculated using the Cochrane ‘Sq test and the I^2^ index (I^2^ >50% indicating high heterogeneity). When there is high heterogeneity among studies, the random effects model is adopted, otherwise the fixed effects model is adopted (29). P value < 0.05 was considered statistically significant. Begg’s method was used to test the publication bias among various studies and to draw a funnel plot. Finally, a sensitivity analysis was performed to determine the impact of individual studies on the aggregated results and to test the reliability of the results.

## 3 Results

### 3.1 Identify eligible studies

**Figure 1:**
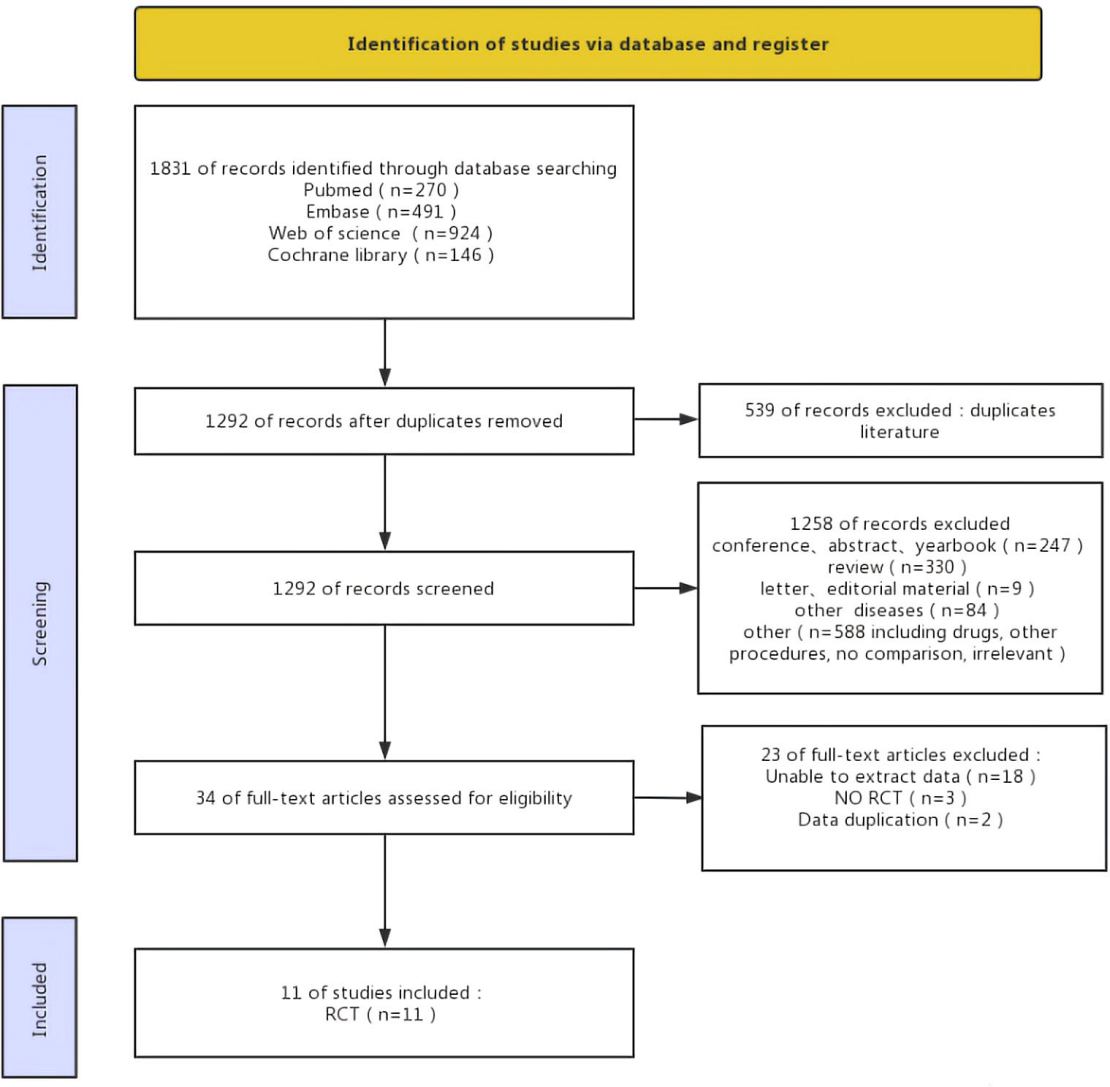
Flow chart of literature search strategies.

The selection process of the study was present in **Figure 1**. A total of 1831 records were retrieved from the four databases and 539 duplicate records were deleted before screening. Then, 1292 records were screened and 1258 were excluded. 34 reports were assessed as qualified and 23 were excluded (unable to extract data =18; Non-rct =3; Data duplication =2). Finally, we included 11 RCTS (**Figure 1**).

### 3.2 Study characteristics

**Table 1** shows the characteristics of the included RCTS. Four studies were from South Korea (30, 31, 35, 39), three from Europe (34, 40, 41), one from Egypt (33), and three from China (32, 36, 37). In these 11 randomized controlled trials, 1,656 participants received RACS and 1,759 received LACS.

**Table 1:** Characteristics of the included studies.

Author, year	country	design	Study Period	group	cases	mean age	Male%	Procedures	Robotic device
**Baik** **2009** (30)	**Korea**	**RCT**	**2006-2007**	**R** **L**	**56** **57**	**60.30** **63.20**	**66.1** **59.6**	**Rectal Cancer**	**Da Vinci** **Surgical System (Intuitive Surgical,California)**
**Kim** **2018** (31)	**Korea**	**RCT**	**2012-2015**	**R** **L**	**66** **73**	**60.40** **59.70**	**77.3** **71.2**	**Rectal Cancer**	**Da Vinci** **Surgical System (Intuitive Surgical,California)**
**Tang** **2020** (32)	**China**	**RCT**	**2016-2018**	**R** **L**	**65** **64**	**55.10** **58.00**	**55.4** **56.2**	**Rectal Cancer**	**Da Vinci** **Surgical System (Intuitive Surgical,California)**
**Debakey** **2018** (33)	**Egypt**	**RCT**	**2015-2017**	**R** **L**	**21** **24**	**60.00** **62.30**	**42.4** **52.4**	**Rectal Cancer**	**Da Vinci** **Surgical System (Intuitive Surgical,California)**
**Jimenez** **2011** (34)	**Spain**	**RCT**	**2008-2009**	**R** **L**	**28** **28**	**68.00** **61.50**	**42.9** **61.0**	**Colorectal cancer resection**	**Da Vinci** **Surgical System (Intuitive Surgical,California)**
**Park** **2019** (35)	**Korea**	**RCT**	**2009-2011**	**R** **L**	**35** **35**	**62.80** **66.50**	**40.0** **45.7**	**Right colectomy**	**Da Vinci** **Surgical System (Intuitive Surgical,California)**
**Qing** **2022** (36)	**China**	**RCT**	**2016-2020**	**R** **L**	**586** **585**	**59.10** **60.70**	**60.8** **60.5**	**Rectal Cancer**	**Da Vinci** **Surgical System (Intuitive Surgical,California)**
**Qing** **2022** (37)	**China**	**RCT**	**2013-2016**	**R** **L**	**174** **173**	**58.2** **59.5**	**62.1** **65.3**	**Rectal Cancer**	**Da Vinci** **Surgical System (Intuitive Surgical,California)**
**Neil** **2018** (38)	**UK**	**RCT**	**2014-2014**	**R** **L**	**237** **234**	**NA**	**67.9** **67.9**	**Rectal Cancer**	**Da Vinci** **Surgical System (Intuitive Surgical,California)**
**Park** **2023** (39)	**Korea**	**RCT**	**2011-2016**	**R** **L**	**151** **144**	**65.5** **67.2**	**64.2** **68.8**	**Rectal Cancer**	**Da Vinci** **Surgical System (Intuitive Surgical,California)**
**David** **2017** (40)	**UK**	**RCT**	**2011-2014**	**R** **L**	**237** **234**	**64.4** **65.5**	**67.9** **67.9**	**Rectal Cancer**	**Da Vinci** **Surgical System (Intuitive Surgical,California)**

R, robot-assisted surgery; L, laparoscopic surgery; NA, not available.

### 3.3 Risk of Bias assessment

**Figure 2:**
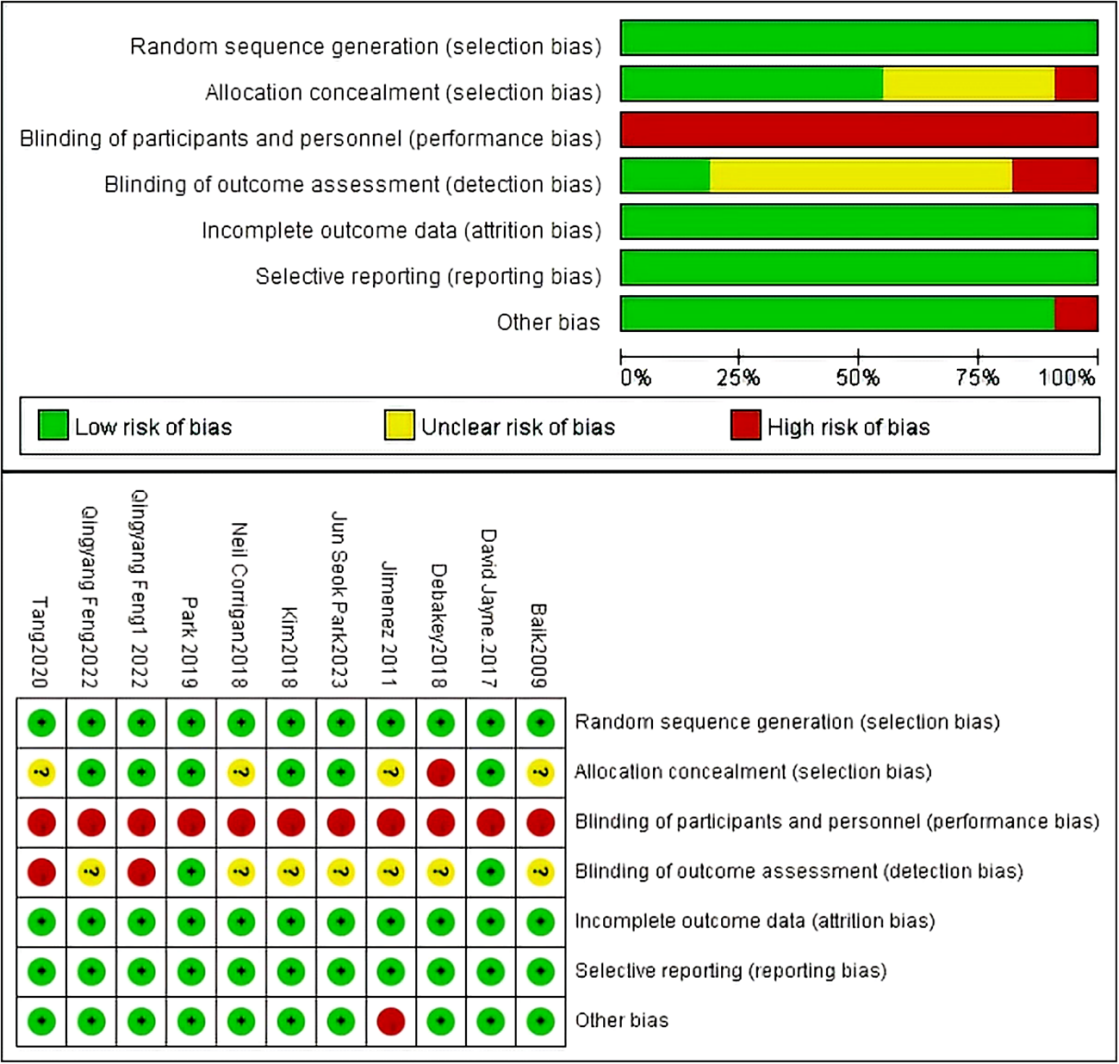
Risk of bias assessment for the included studies.

The results of the risk of bias assessment are summarized in **Figure 2**. Among the 11 studies, an adequate randomized sequence was generated in 11 studies, appropriate allocation concealment was reported in 6 studies, the blinding of participants was clear in no study, the blinding of outcome assessors was reported in 2 studies, outcome data were complete in 11 studies, 11 studies had no selective reporting, and 10 studies had no other bias (**Figure 2**).

### 3.4 Clinical outcomes

All results of the meta-analysis for clinical outcomes were summarized in **Table 2**.

**Table 2:** Results of the meta-analysis.

Outcomes	No. of studies	Sample size		Heterogeneity		Overall effect size	95% CI of overall effect	P Value
		R	L	I^2^(%)	P Value			
Operation time (min)	9	1384	1382	95	<0.00001	WMD=5.19	18.00,39.82	<0.00001
Length of stay (days)	9	1247	1249	95	<0.00001	WMD=2.97	-1.60,–0.33	0.003
Conversion	10	1590	1583	0	0.63	RR=3.62	0.40,0.76	0.0003
Complications	11	1418	1413	26	0.21	RR=3.31	0.64,0.89	0.0009
CRM+	6	1120	1105	0	0.81	RR=1.94	0.49,1.00	0.05
Proximal resection margin(cm)	7	1082	1084	95	<0.00001	WMD=-1.14	-1.16,0.31	0.25
Distal resection margin(cm)	6	908	911	92	<0.00001	WMD=2.16	0.04,0.94	<0.00001
Blood loss(ml)	7	1098	1098	97	<0.00001	WMD=2.71	-33.24,-5.35	0.007
The number of harvested lymph nodes	9	1391	1389	79	<0.00001	WMD=1.70	-0.09,1.31	0.09
The time of first flatus(days)	7	996	1004	99	<0.00001	WMD=0.88	-0.59,0.23	0.38
The time of first autonomous urination(days)	4	853	850	99	<0.00001	WMD=1.59	-2.01,0.21	0.11
The time of first defecation(days)	2	652	658	64	0.10	WMD=1.44	-0.80,0.12	0.15
The time of first resume diet(days)	6	975	980	97	<0.00001	WMD=1.77	-0.82,0.04	0.08
Reoperation rates	4	816	817	0	0.96	RR=2.12	0.33,0.96	0.03
Readmission rates	4	816	817	4	0.37	RR=1.45	0.41,1.14	0.15
Death rates	5	1083	1080	0	0.91	RR=0.97	0.24,1.62	0.33
Quality of TME:Complete	8	1356	1354	5	0.39	RR=2.76	1.01,1.08	0.006
Quality of TME:Incomplete	8	1356	1354	55	0.03	RR=2.53	0.67,0.95	0.01

#### 3.4.1 Operative time (min)

**Figure 3:**
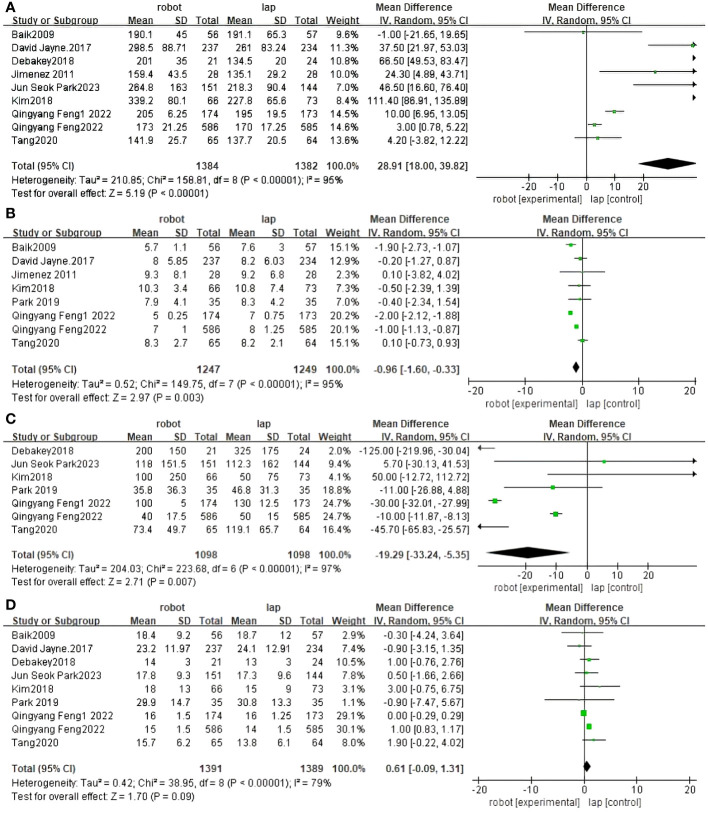
Forest plot of the meta-analysis for clinical outcomes. **(A)** Operative time. **(B)** Length of stay. **(C)** Blood loss. **(D)** The number of harvested lymph nodes.

Operative time was reported in nine RCTs (30–37, 40). The pooled results showed a significant difference between RACS and LACS, with LACS having a shorter surgical time than RACS(MD=5.19,95%CI:18.00,39.82, P<0.00001;I ^2 =^ 95%, P_Q_<0.00001) (**Figure 3A**).

#### 3.4.2 Length of stay (days)

Length of stay was reported in eight RCTs (30–32, 34, 36, 37, 39, 40). The difference between RACS and LACS was statistically significant, with RACS having a shorter hospital stay than LACS.(MD=2.97,95%CI:−1.60,−0.33, P = 0.003;I ^2 =^ 95%;P_Q_<0.00001) (**Figure 3B**).

#### 3.4.3 Blood loss (ml)

Seven randomized controlled trials reported blood loss between RACS and LACS (31–33, 35–37, 39). There was a significant difference between RACS and LACS, with RACS having lower blood loss than LACS(MD=2.71,95%CI:−33.24,−5.35, P = 0.007;I 2 = 97%, P_Q_< 0.00001) (**Figure 3C**).

#### 3.4.4 The number of harvested lymph nodes

Nine randomized controlled trials reported the number of lymph nodes harvested between RACS and LACS (30–33, 35–37, 39, 40). There was no statistically significant difference between RACS and LACS(MD=1.70,95%CI:−0.09,1.31,P=0.09;I ^2 =^ 79%,P_Q_< 0.00001) (**Figure 3D**).

#### 3.4.5 Conversion to other surgery

**Figure 4:**
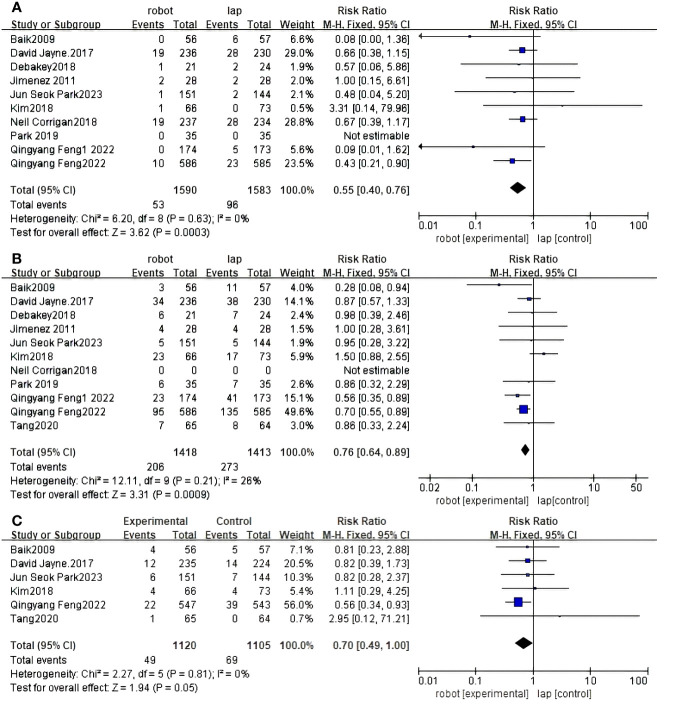
Forest plot of the meta-analysis for clinical outcomes. **(A)** Conversion. **(B)** Complications. **(C)** CRM+.

Ten RCTS reported conversion rates for open surgery between RACS and LACS (30, 31, 33–37, 39–41). There is a significant difference between RACS and LACS, and the conversion rate of RACS is lower(RR=3.62,95%CI:0.40,0.76,P = 0.0003;I ^2 =^ 0%,P_Q_=0.63) (**Figure 4A**).

#### 3.4.6 Complications

Eleven RCTS reported complication rates between RACS and LACS (30–37, 39–41). There was a significant difference in the complication rate between RACS and LACS, with RACS having a lower complication rate(RR=3.31,95%CI:0.64,0.89,P = 0.0009;I ^2 =^ 26%,P_Q_=0.21) (**Figure 4B**).

#### 3.4.7 CRM+

Six studies showed CRM+ (30–32, 35, 36, 40) rates; RACS and LACS had no significant difference in the occurrence of CRM+(RR=1.94, 95%CI:0.49,1.00,P = 0.05;I ^2 =^ 0%,P_Q_=0.81) (**Figure 4C**).

#### 3.4.8 Proximal resection margin (cm)

**Figure 5:**
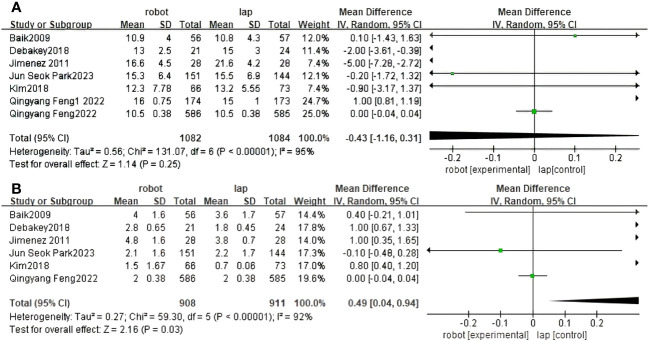
Forest plot of the meta-analysis for clinical outcomes. **(A)** Proximal resection margin. **(B)** Distal resection margin.

Seven studies reported the Proximal resection margin of RACS and LACS (30, 31, 33–37). There were no significant differences between RACS and LACS(MD=1.14, 95%CI:-1.16,0.31,P = 0.25;I ^2 =^ 95%,P_Q_<0.00001) (**Figure 5A**).

#### 3.4.9 Distal resection margin (cm)

Distal resection margin of RACS and LACS was reported in six studies (30, 31, 33–36). There were significant differences between RACS and LACS. RACS improved the distal incisal margin better than LACS(MD=2.16, 95%CI:0.04,0.94,P = 0.03;I ^2 =^ 92%,P_Q_<0.00001) (**Figure 5B**).

#### 3.4.10 The time of first flatus (days)

**Figure 6:**
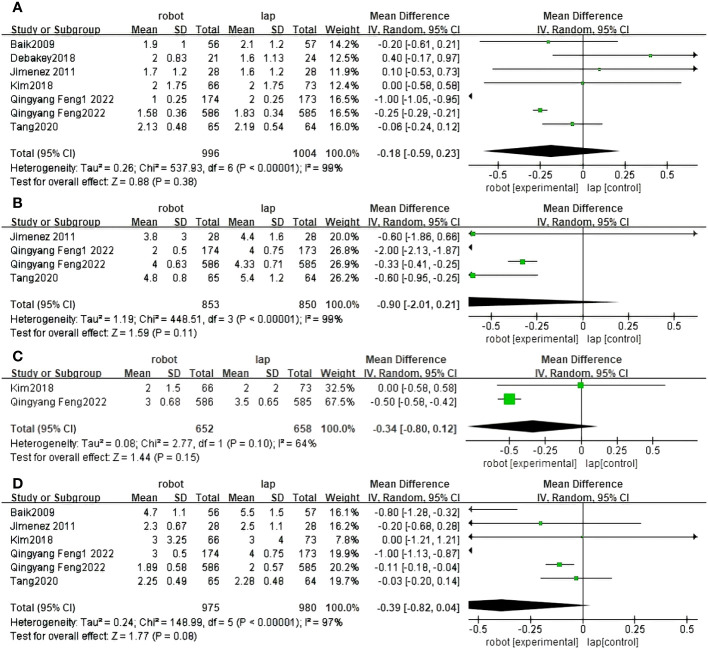
Forest plot of the meta-analysis for clinical outcomes. **(A)** Time of first flatus. **(B)** Time of first autonomous urination. **(C)** Time of first defecation. **(D)** Time of first resume diet.

Seven randomized controlled trials reported first exhaust time between RACS and LACS (30–34, 36, 37). The difference between RACS and LACS was not statistically significant (MD=0.88,95%CI:−0.59,0.23,P = 0.38;I ^2 =^ 99%,P_Q_< 0.00001) (**Figure 6A**).

#### 3.4.11 The time of first autonomous urination (days)

Four randomized controlled trials reported first urination days between RACS and LACS (32, 34, 36, 37). There was no statistically significant difference between RACS and LACS(MD=1.59, 95%CI:−2.01,0.21,P=0.19;I ^2 =^ 99%,P_Q_< 0.00001) (**Figure 6B**).

#### 3.4.12 The time of first defecation (days)

Two randomized controlled trials reported first defecation days for RACS and LACS (31, 36). There was no statistically significant difference between RACS and LACS(MD=1.44, 95%CI:−0.80,0.12,P=0.15;I ^2 =^ 64%,P_Q_= 0.10) (**Figure 6C**).

#### 3.4.13 The time of first resume diet (days)

Six studies reported the time to resume diet between RACS and LACS (30–32, 34, 36, 37). There was no statistically significant difference between RACS and LACS(MD=1.77,95%CI:−0.82,0.04, P=0.08;I ^2 =^ 97%, P_Q_< 0.00001) (**Figure 6D**).

#### 3.4.14 Reoperation rates

**Figure 7:**
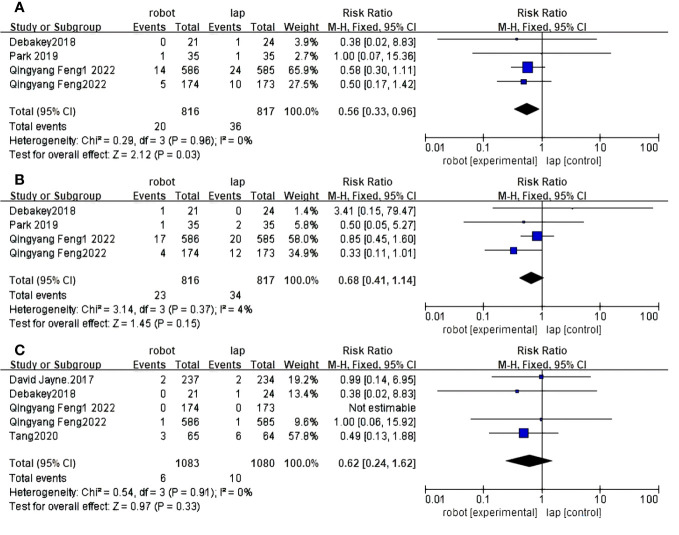
Forest plot of the meta-analysis for clinical outcomes. **(A)** Reoperation rates. **(B)** Readmission rates. **(C)** Death rates.

Four studies reported reoperation rates between RACS and LACS (33, 36, 37, 39). The difference between RACS and LACS was statistically significant, and the reoperation rate of RACS was lower than that of LACS(RR=2.12, 95%CI:0.33,0.96,P=0.03;I ^2 =^ 0%,P_Q_= 0.96) (**Figure 7A**).

#### 3.4.15 Readmission rates

Four randomized controlled trials reported readmission rates between RACS and LACS (33, 36, 37, 39). There was no statistically significant difference between RACS and LACS(RR=1.46, 95%CI:0.41,1.14,P=0.15;I ^2 =^ 4%,P_Q_= 0.37) (**Figure 7B**)

#### 3.4.16 Death rates

Five randomized controlled trials reported mortality between RACS and LACS (32, 33, 36, 37, 40), with no statistically significant difference between RACS and LACS(RR=0.97,95%CI:0.24,1.62,P=0.33;I ^2 =^ 0%,P_Q_=0.91) (**Figure 7C**).

#### 3.4.17 Complete rates of TME

**Figure 8:**
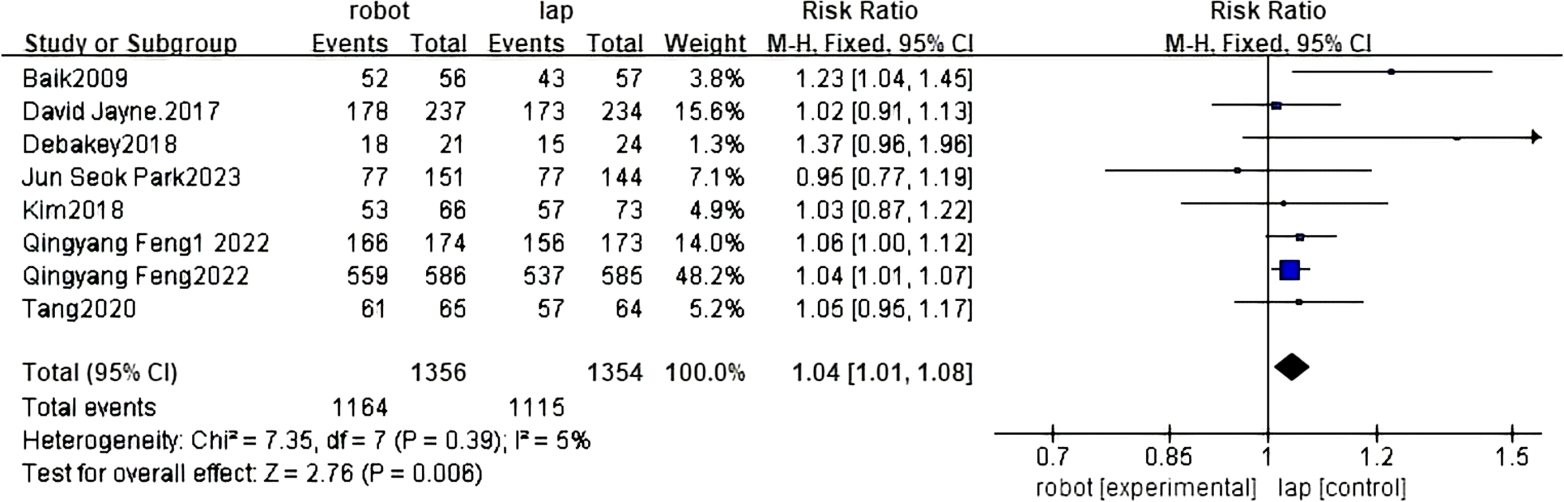
Forest plot of the meta-analysis for complete rates of TME.

Eight studies have reported the integrity of total mesorectal resection of RACS and LACS (30–33, 35–37, 40). There was statistical significance between RACS and LACS, and the complete resection rate of RACS was higher(RR=2.76, 95%CI:1.01,1.08,P=0.006;I ^2 =^ 5%,P_Q_=0.39) (**Figure 8**).

### 3.5 Sensitivity analysis

Sensitivity analysis was performed on conversion rate, complication rate, CRM+ rate and number of lymph node dissection (**Supplementary Material**). Sensitivity analysis shows that the results of conversion rate and CRM+ rate are robust. The sensitivity analysis was carried out by excluding literatures one by one. Although the results of complication incidence changed, the results were still robust from the whole point of view. The sensitivity analysis of the number of lymph node dissection was carried out, and the results were all changed after deleting the literatures. After observing the data changes, the literature (37) with the greatest difference in selective changes was removed one by one, and the results showed no change, indicating that the heterogeneity came from this literature.

### 3.6 Publication bias

**Figure 9:**
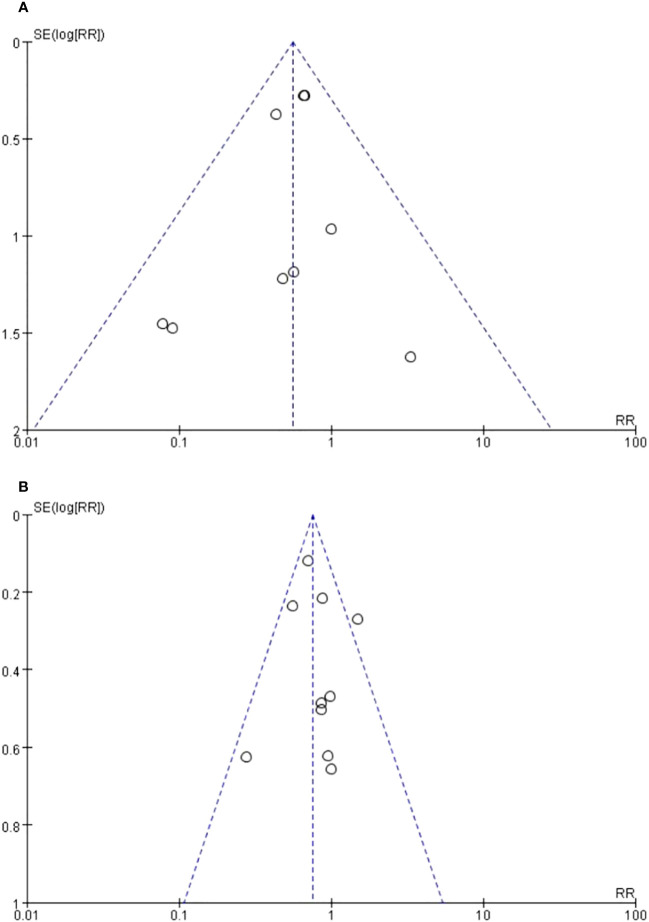
Funnel plot. **(A)** Conversion rates. **(B)** complication rates.

The main indicators in this review included conversion rates and complication rates. Eleven randomized trials reported complication rates and ten studies reported conversion rates. Funnel plots were conducted to examine the presence of significant publication bias. Bilateral symmetric funnel plots of conversion rates show that no significant evidence of publication bias is observed (**Figure 9A**). Bilateral symmetric funnel plots of complication rates showed that no significant evidence of publication bias was observed (**Figure 9B**).

## 4 Discussion

Colorectal resection is considered as the gold standard treatment for colorectal cancer (8–10). In recent year, as a relatively new platform for minimally invasive surgery, RACS has been proposed as an alternative to LACS (12). However, previous studies comparing the clinical outcomes of RACS with LACS has not been sufficient to prove the benefits of RACS. Some of previous reviews and meta-analysis included non-randomized and observational studies in the meta-analysis which posed a risk of bias (23, 25, 38). Another meta-analysis included only RCTs, but only six studies was selected, leading to a relatively small number of patients and limited outcomes (24). In the present study,11 RCTs was included, and a high quality meta-analysis was conducted to compare outcomes of RACS versus LACS in the treatment of colorectal cancer.

According to the present meta-analysis, RACS reduces complication rates, blood loss, conversion rates, reoperation rates and hospital stay, and provides better distal margin results as compared to the LACS cohort. Previous study has reported similar results (18–22, 38). RACS has several advantages over LACS. Different from the four-degrees-of-freedom instruments in LACS, the seven-degrees-of-freedom robotic arms in RACS allow surgeons to perform more meticulous and precise procedure (42). Besides, high-quality 3-dimensional imaging with magnification, better ergonomic, stable platform of camera controlled by surgeons and free moving multi-joint forceps were provided in RACS (43). In mininvasive surgery, the delicate handling of RACS provides safer surgical procedure and more efficient tumor resection compared with LACS (44). Multi-articulated instrument of RACS allows surgeons to carefully manipulate the blood vessels, rapidly control and minimize bleeding (45).

Our results declared that RACS has a longer surgical duration than LACS. This is likely due to several factors including of docking time, more technically demanding procedures like intracorporeal suturing and learning curve (46). Previous studies (25, 38, 47–49)reported that RACS had longer operation time compared to LACS, which is consistent with our results (38, 48, 49). With regard to surgical time, along with surgeons becoming more familiar with the robot, the learning curve decreases and the differences seen will gradually balance out (50, 51). Rausa et al. (52) reported that surgery time could be influenced by a surgeon’s learning curve, and the operative time in RACS became similar to that of LACS in right-sided hemicolectomy for cancer after 21 cases.

In terms of harvested lymph nodes, time of first flatus, time of first defecation, time of first resume diet,proximal resection margin, readmission rates, mortalities and CRM+ rates, our study reported no statistical difference between RACS and LACS. In theory, longer surgery times are associated with harmful outcomes and may lead to longer hospital stays and increased conversion rates, but previous studies have shown lower conversion rates (38, 50, 53) and shorter hospital stays (38, 54, 55). Several studies have reported that robotic surgery produces similar perioperative outcomes to conventional laparoscopic surgery (56, 57). In addition, some previous studies have also shown that compared with LACS, RACS has less blood loss, fewer complications, and lower mortality, bleeding and intestinal obstruction rates (55).

To our knowledge, the present meta-analysis included the largest number of randomized controlled trials comparing outcomes of RACS versus LACS in the treatment of colorectal cancer, which could result in relatively robust conclusion. Besides, supplementary sensitivity analyses were performed to strengthen our results and overcome the risk of baseline confounding regarding short-term outcomes presenting with high heterogeneity, supporting our findings from the primary analysis. The findings of our study provide valuable insights into the clinical outcomes of colorectal surgical approaches which contribute to clinical practice and research. However, we acknowledge the possible limitations of our study. First of all, we failed to control the confounding factors such as the type of colorectal procedures, the level of expertise of surgeons involved and total versus hybrid robotic surgery. Though Da Vinci Surgical System (Intuitive Surgical, California) was used in all these trials (**Table 1**), we failed to identify if there were different models of the same device, which might be considered as a bias. Second, long-term outcomes such as 5-year overall survival were not analyzed because of the short follow-ups of the studies included. Third, trials published as abstract or presented at conferences were removed, which may potentially introduce publication bias to our findings. Forth, the number of RCTs included was still relatively small due to the strictest criteria, which result in a relatively small number of patients, and the impact of RACS may be overestimated compared to studies with large samples.

In conclusion, our study indicated that RACS is a feasible and safe technique that can achieve better surgical efficacy compared with LACS in terms of short-term outcomes. Except of longer operation time, RACS has obvious advantage in hospital stay, conversion rate, complication rate, blood control, reoperation rate and distal margin results. However, large sample and long follow-up randomized clinical trials comparing RACS with LACS are still necessary to better demonstrate the advantages of RACS for colorectal cancer.

## Data availability statement

The original contributions presented in the study are included in the article/**Supplementary Material.** Further inquiries can be directed to the corresponding author.

## Author contributions

WL: Funding acquisition, Writing – original draft, Writing – review & editing. ZH: Data curation, Writing – original draft. SH: Data curation, Formal Analysis, Writing – original draft. YH: Conceptualization, Investigation, Writing – original draft. RL: Methodology, Visualization, Writing – original draft.

## Supplementary material

Supplementary Table 1 The details of the searching record in Medline.

Supplementary Table 2 The details of the searching record in CENTRAL.

Supplementary Table 3 The details of the searching record in Embase.

Supplementary Table 4 The details of the searching record in Web of Science.
