# Supplementary material for: Comparison of robotic-assisted versus conventional laparoscopic surgery in colorectal cancer resection: a systemic review and meta-analysis of randomized controlled trials
Source: Front Oncol. 2023 Oct 26;13:1273378. doi: 10.3389/fonc.2023.1273378 (PMC10641393; doi:10.3389/fonc.2023.1273378)

Complication rates


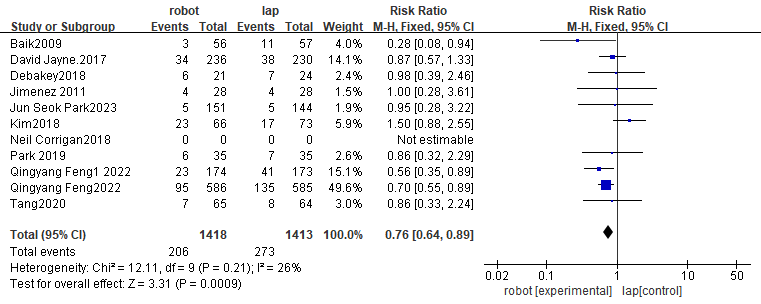


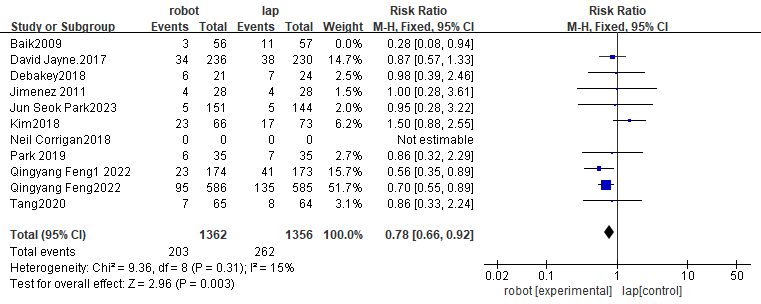


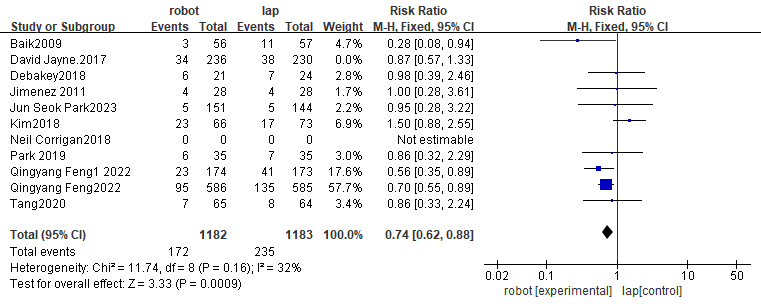


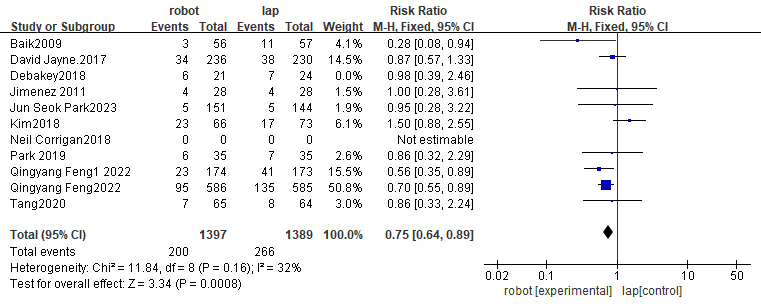


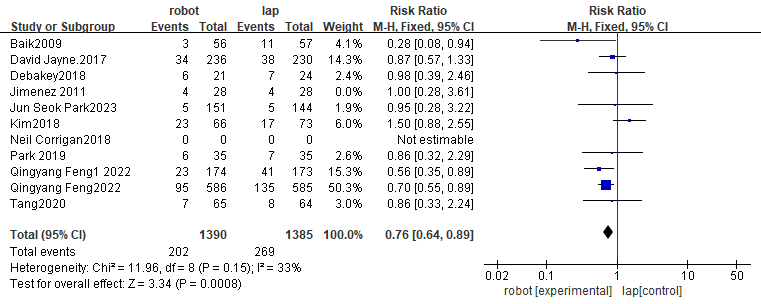


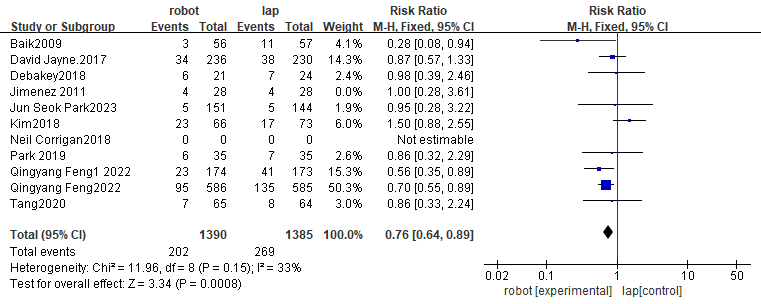


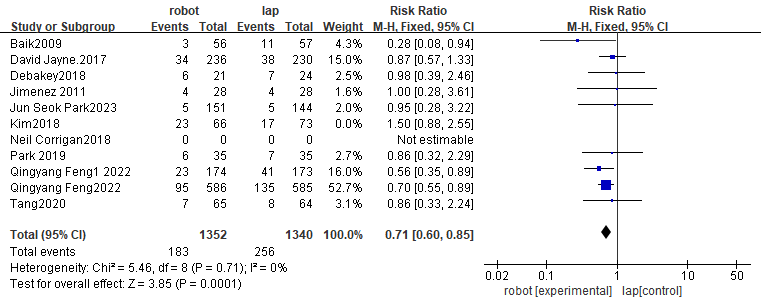


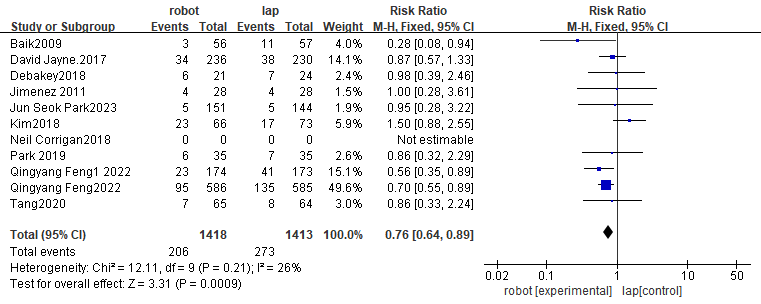


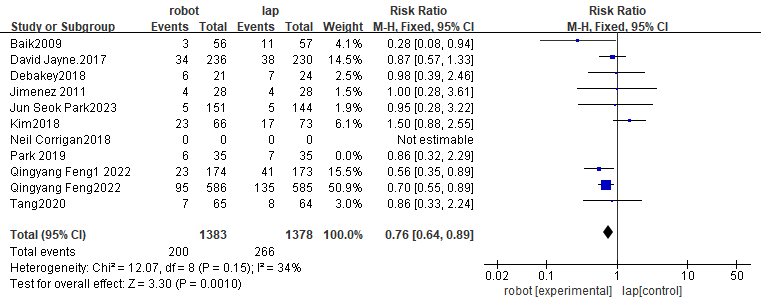

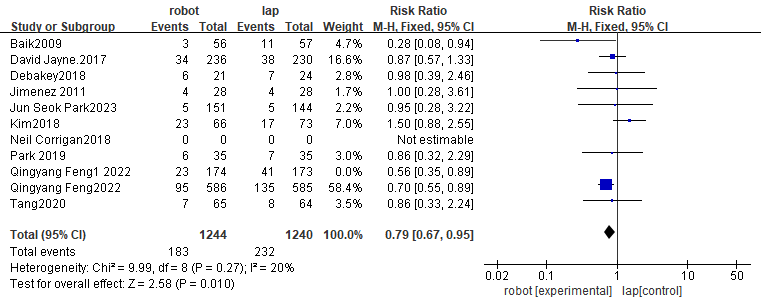

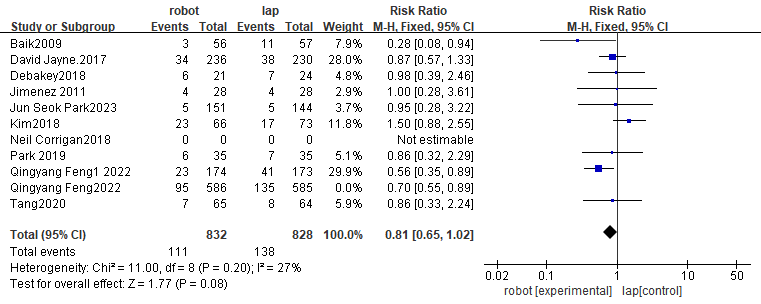

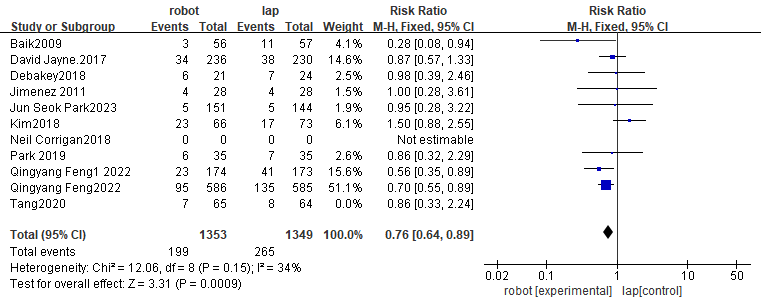


Conversion rates


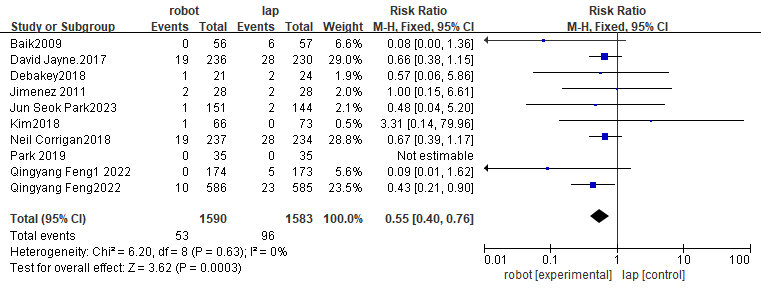

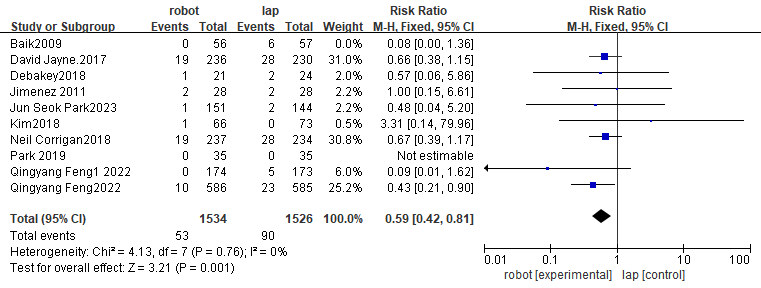

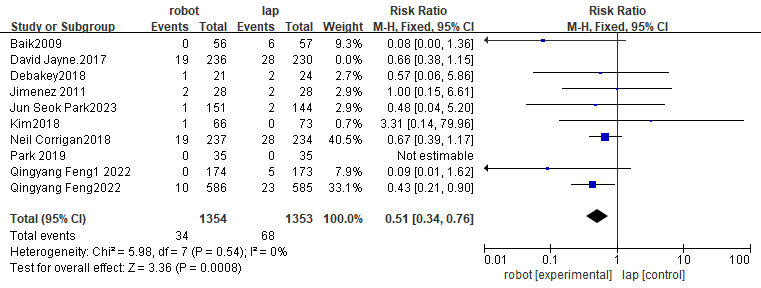

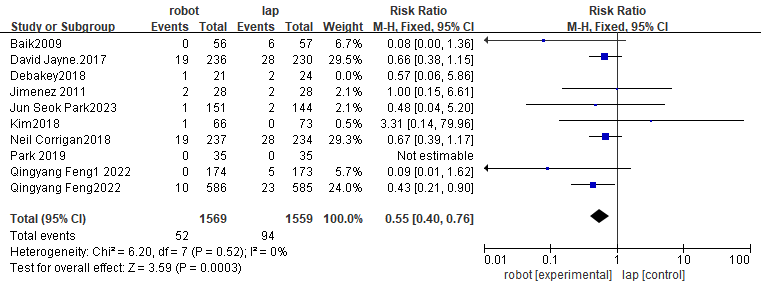

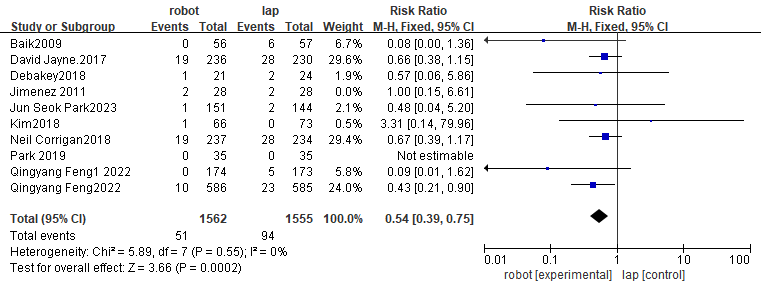

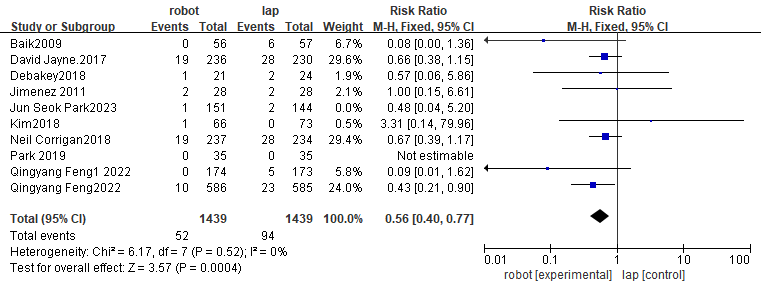

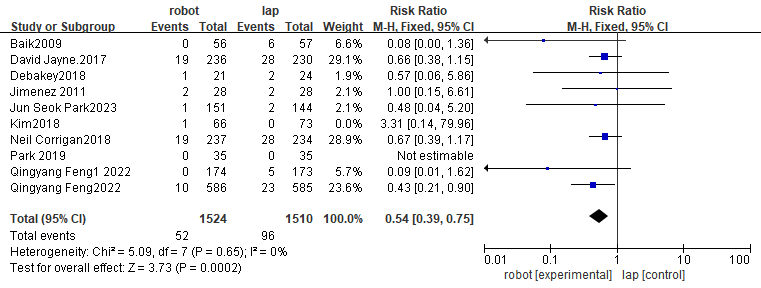

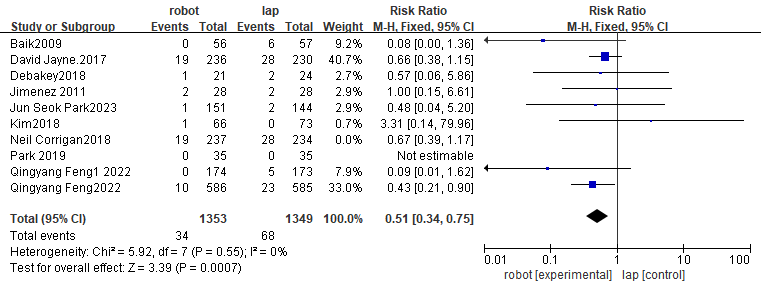

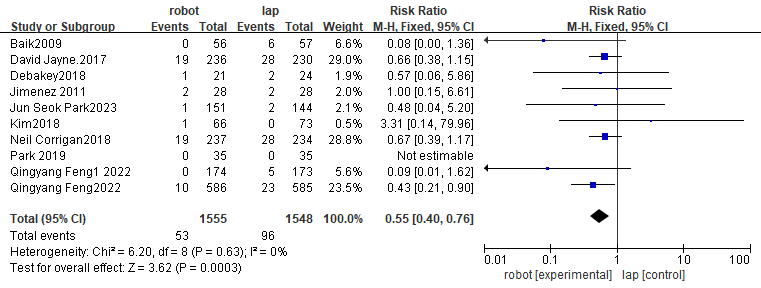

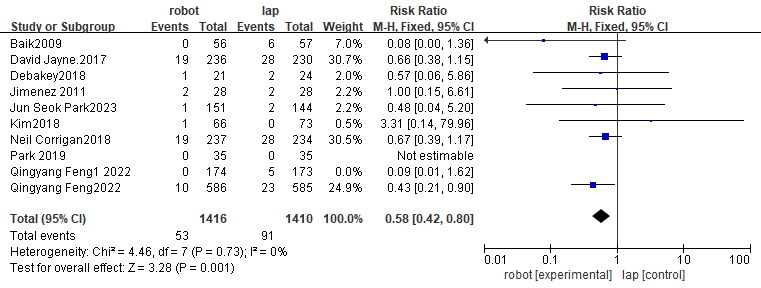

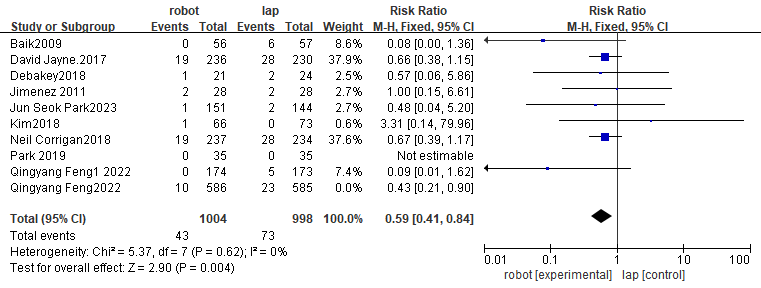


CRM+ rates


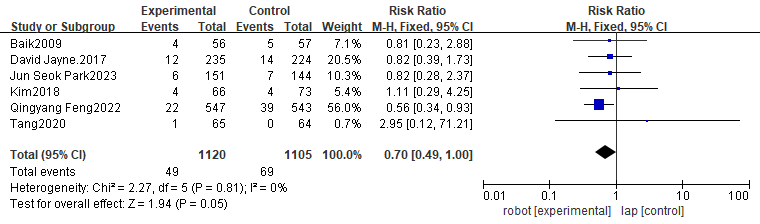

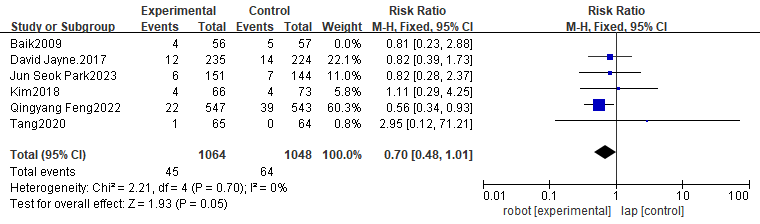

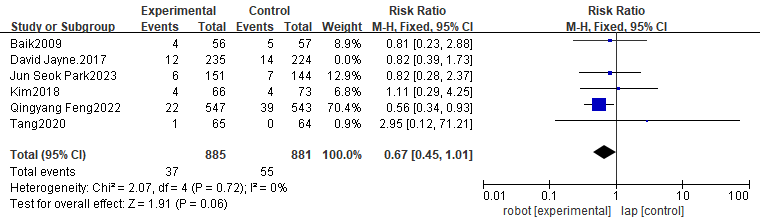

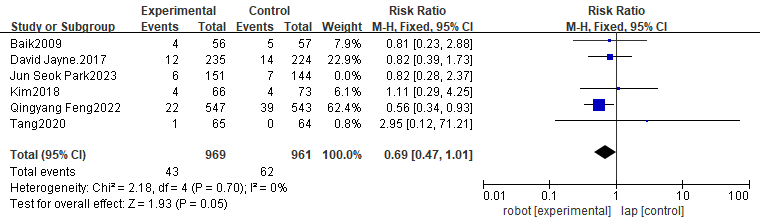

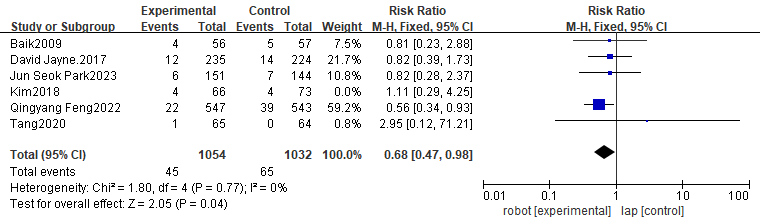

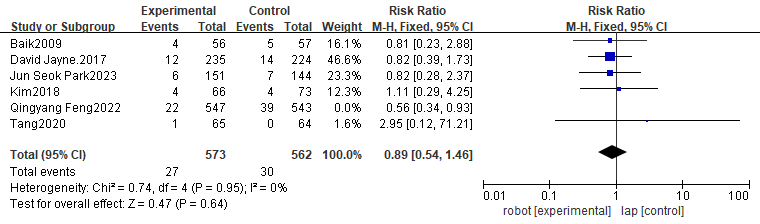

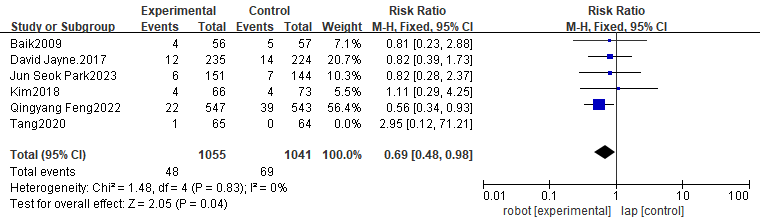


Harvested lymph nodes


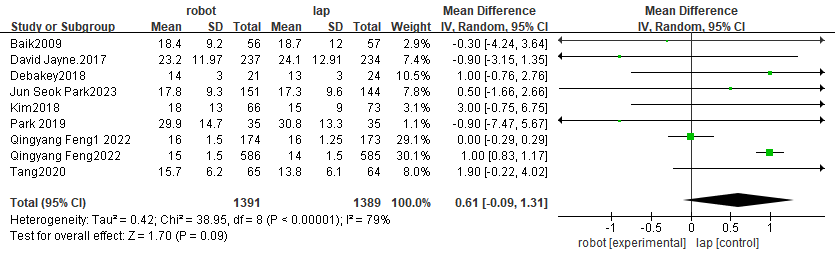

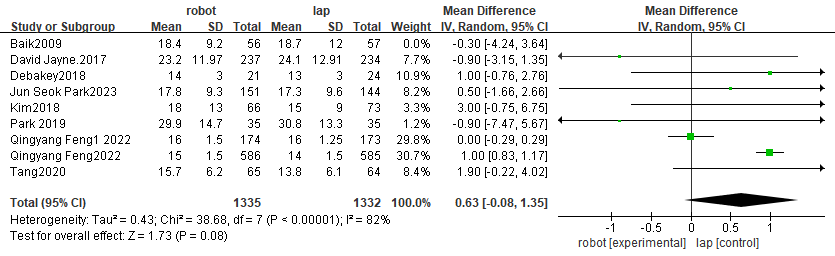

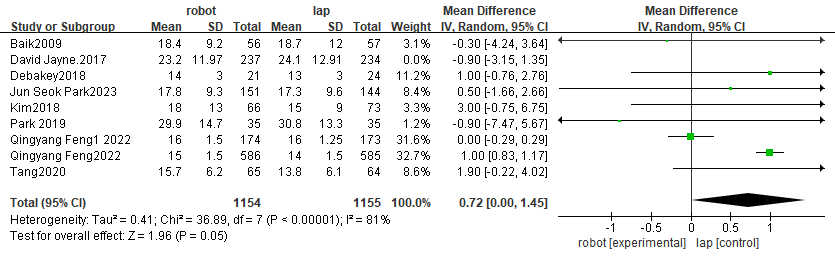

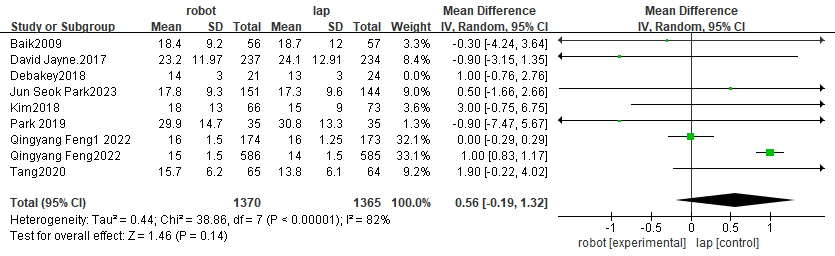

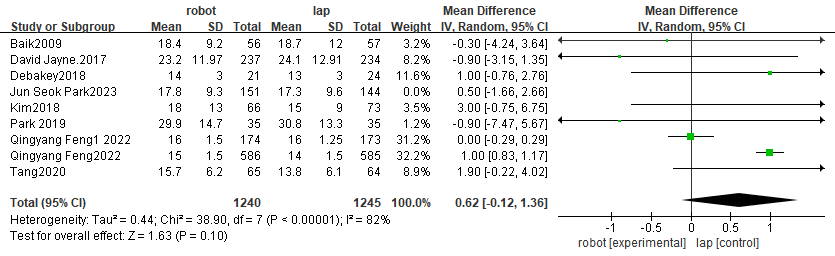

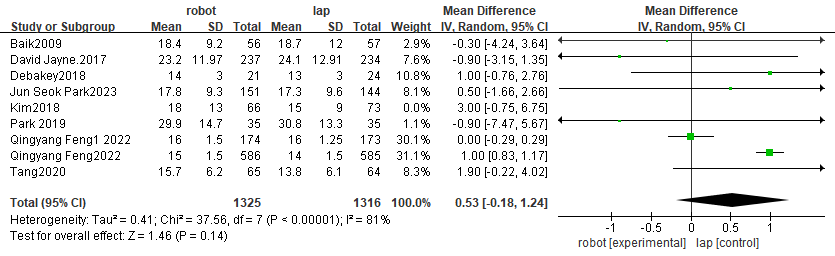

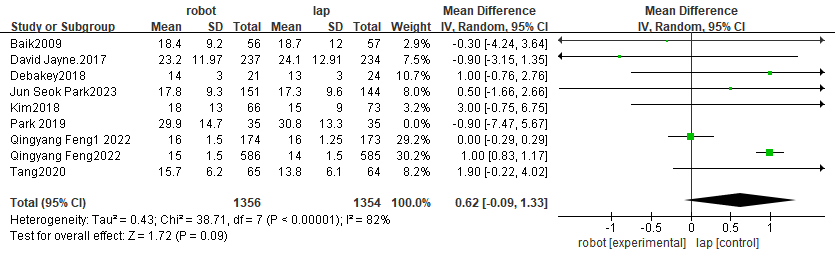

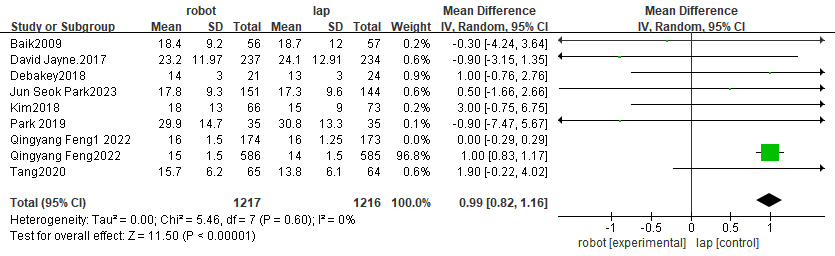

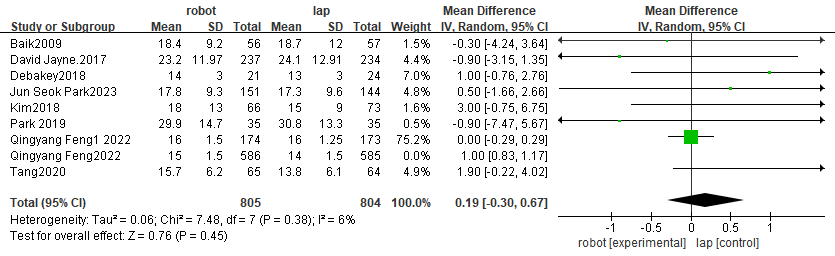

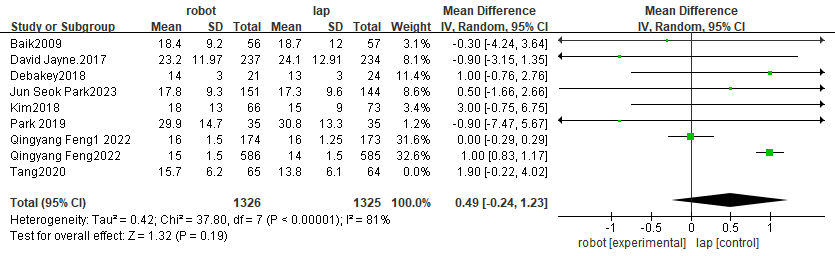


Exclude Qingyang Feng1 2022，then exclude the other documents one by one


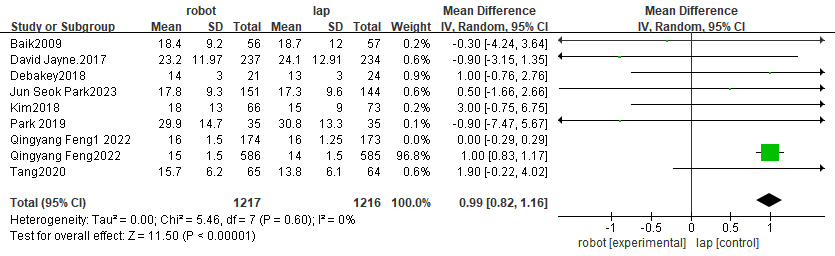

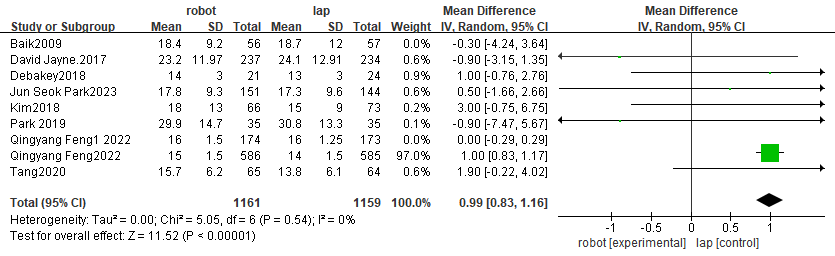

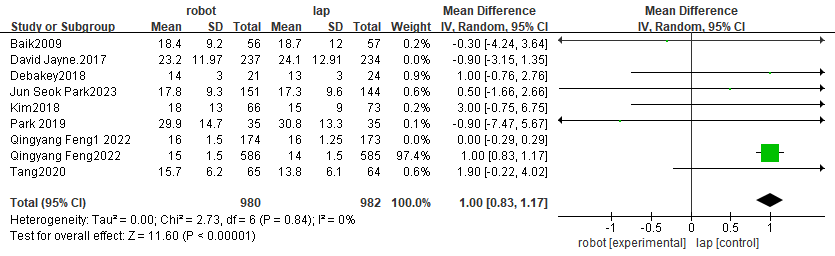

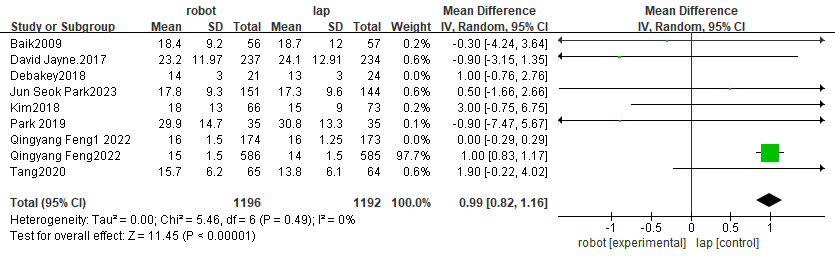

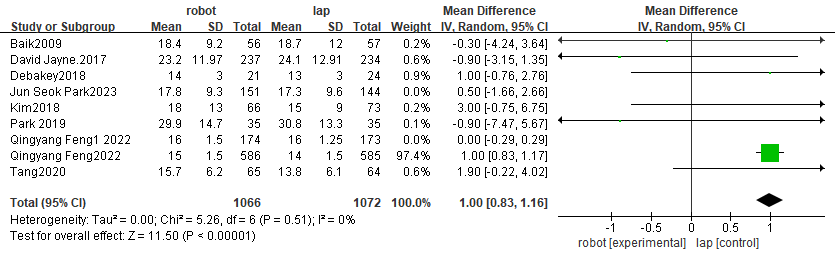

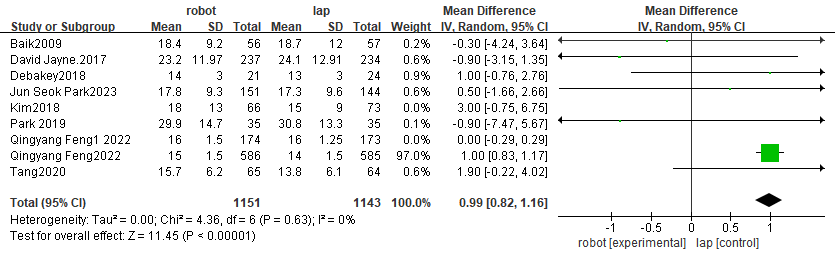

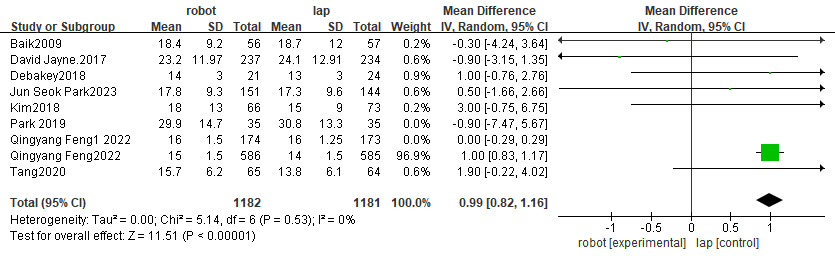

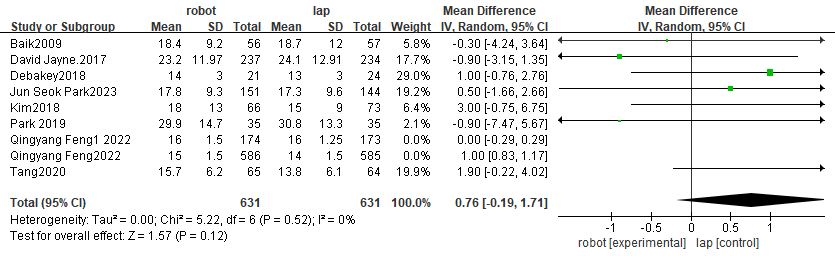

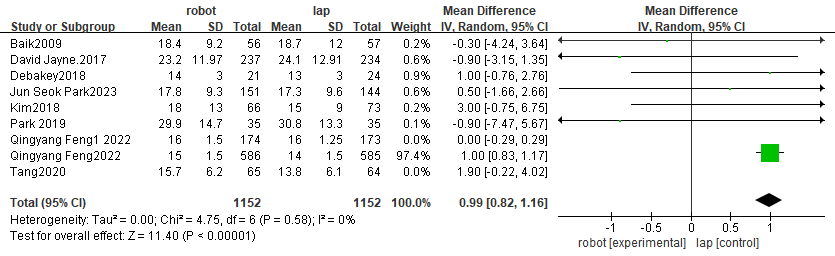

Supplement: Supplementary Table 1 — The details of the searching record in Medline. [file DataSheet_1.docx]
